# Transcriptomic and Proteomic Analyses of the Immune Mechanism in Pathogenetic and Resistant Chinese Soft-Shelled Turtle (*Pelodiscus sinensis*) Infected with *Aeromonas hydrophila*

**DOI:** 10.3390/genes15101273

**Published:** 2024-09-27

**Authors:** Lingrui Ge, Zi’ao Wang, Yazhou Hu, Pei Wang, Qin Qin, Yu Tian, Xiaoqing Wang, Xingxing Wen, Dan Zeng

**Affiliations:** 1 College of Animal Science and Technology, Hunan Biological and Electromechanical Polytechnic, Changsha 410127, China; gelingrui@hnbemc.edu.cn (L.G.); 13739069939@163.com (Z.W.); 2 Fisheries College, Hunan Agricultural University, Changsha 410128, China; huyazhou@hunau.edu.cn (Y.H.); qinqin880404@hotmail.com (Q.Q.); tianyu_988426@163.com (Y.T.); wangxiao8258@126.com (X.W.); 3 College of Biology and Environmental Sciences, Jishou University, Jishou 416000, China; wangpei0229@126.com; 4 College of Life and Environmental Sciences, Hunan University of Arts and Science, Changde 415000, China

**Keywords:** *Pelodiscus sinensis*, *Aeromonas hydrophila*, transcriptomic, proteomic, immune mechanism

## Abstract

Background: As intensive aquaculture practices have progressed, the prevalence of bacterial diseases in the Chinese soft-shell turtle (*Pelodiscus sinensis*) has escalated, particularly infections caused by *Aeromonas hydrophila*, such as ulcerative dermatitis and abscess disease. Despite this, little is known about their immune defenses against this pathogen. Methods: Our study pioneers an integrated analysis of transcriptomics and proteomics to investigate the immune responses of Chinese soft-shelled turtles to *A. hydrophila* infection. Results: The investigation revealed significant differences in immune-related pathways between groups susceptible and resistant to *A. hydrophila* infection after 4 days. A total of 4667 and 3417 differentially expressed genes (DEGs), 763 and 568 differentially expressed proteins (DEPs), and 13 and 5 correlated differentially expressed genes and proteins (cor-DEGs-DEPs) were identified in susceptible and resistant Chinese soft-shelled turtles, respectively. In the resistant group, upregulation of immune-related genes, such as CD3ε and CD45, enhanced T-cell activation and the immune response. The proteomic analysis indicated that immune proteins, such as NF-κB1, were significantly upregulated in the resistant group. The correlation analysis between transcriptomics and proteomics demonstrated that the CD40 gene and protein, differentially expressed in the resistant group compared to the control group, were commonly upregulated within the Toll-like receptor signaling pathway. Conclusions: The transcriptomic and proteomic data obtained from this study provide a scientific foundation for understanding the immune mechanisms that enable the Chinese soft-shelled turtle to resist *A. hydrophila* infection.

## 1. Introduction

The Chinese sSoft-shelled turtle (*Pelodiscus sinensis*), a benthic aquatic animal that inhabits freshwater environments, is a high-protein, low-fat aquatic product of great commercial value in China [1,2]. The escalating intensity of aquaculture has given rise to a predicament wherein the carrying capacity of ponds falls short of meeting production demands, subsequently triggering a cascade of persistent challenges. This has compromised the delicate balance between physiological processes and microbial ecosystems [3], ultimately precipitating a surge in the incidence of diseases afflicting the Chinese soft-shelled turtle. This escalation has led to pronounced mortality rates, imposing substantial economic burdens on the aquaculture community, with bacterial infections emerging as the primary threat [4]. Among these, *Aeromonas hydrophila* has been pinpointed as the causative agent for 15 prevalent bacterial ailments [5], encompassing ulcerative dermatitis [6] and white abdominal shell disease [7]. Despite these formidable challenges, a comprehensive understanding of the molecular mechanisms underlying the Chinese soft-shelled turtle’s response to *A. hydrophila* infection remains elusive. Furthermore, the development of a robust, integrated strategy for prevention and control is still a work in progress. Thus, a meticulous exploration of the turtle’s immunological defenses against this pathogen, coupled with the enhancement of its immune resilience, not only carries profound economic implications but also holds vital practical significance. Such endeavors are instrumental in bolstering the efficacy of artificial cultivation practices and ensuring the sustainability of the aquaculture industry.

In recent years, transcriptomics, proteomics, and their integrated analysis have been widely used in the study of aquatic animals. They help us to understand the genetic background, gene expression and regulation, and protein expression of aquatic organisms, and explore the interaction between the immune system of aquatic animals and pathogen infection, revealing the complex molecular mechanisms of host–pathogen interactions [4]. The application of transcriptomics in the immune field of aquatic animals mainly includes the study of the differential expression of immune-related genes before and after animal infection with microorganisms [8] or parasites [9], before and after changes in feed components [10,11,12,13], and before and after vaccine use [14]. Jiang Baijian et al. [8] found through transcriptomic analysis that the TOX4 gene in Nile tilapia is widely expressed in various tissues in a healthy state, with the highest expression in the blood. After infection with Streptococcus agalactiae, the expression of TOX4 in the brain, head kidney, intestine, and spleen significantly increased, especially peaking at 12 h (brain, head kidney, intestine) and 48 h (spleen) after infection, indicating that TOX4 may play an important role in the antibacterial defense of *Nile tilapia.*

The application of proteomics in the immunology of aquaculture animals is also very important and is primarily used to analyze the physiological adaptation mechanisms of aquaculture animals under changes in nutritional status and environmental conditions, especially the response of aquaculture animals to bacteria [15], viruses [16,17,18], and other aquatic environmental stresses [19]. This facilitates a deeper comprehension of the immunological and pathogenetic mechanisms within aquaculture hosts, along with the intricate interplay between the host and the pathogen [20,21]. Lü et al. [19] conducted a proteomic analysis on the skin of zebrafish infected with *A. hydrophila* and found that the expressions of actin, myosin heavy chain, and GAPDH increased. Furthermore, some studies have shown that actin plays an important role in resisting bacterial infections [22].

A combined analysis of transcriptomics and proteomics can provide a more comprehensive understanding of the mechanisms involved from multiple levels. For instance [23], Li et al. [24] conducted a transcriptomic and proteomic analysis of grass carp intestine after vaccination with a bivalent DNA vaccine against *Vibrio mimicus*. The results showed differences in the expression levels of genes and proteins related to phagocytosis, antigen processing and presentation, the complement system, and coagulation cascades, indicating that these processes are crucial for enhancing the mucosal immune response in the intestine of immunized fish.

The spleen, an essential immune organ in animals, plays a crucial role in immune responses to pathogen infections. An in-depth study of the immune mechanisms within the Chinese soft-shelled turtle’s spleen, particularly in response to *A. hydrophila* infection, utilizes a combined analysis of transcriptomics and proteomics. This approach establishes a foundation for further elucidation of the molecular mechanisms behind immune-related genes and proteins in the Chinese soft-shelled turtle’s disease resistance process. It also offers valuable theoretical insights for the rational prevention and treatment of diseases, as well as for investigating the pathogenetic mechanisms of the Chinese soft-shelled turtle.

## 2. Materials and Methods

### 2.1. Experimental Animals and Sample Collection

Six hundred healthy juvenile Chinese soft-shelled turtles (mean weight: 20.0 ± 0.5 g) were randomly selected from Changde Hezhou Aquatic Products Co., Ltd., Changde, China. All the animal studies adhered to protocols approved by the Animal Care Advisory Committee of Hunan Biological And Electromechanical Polytechnic. The turtles were acclimatized for two weeks in a recirculating aquaculture system, during which they were fed daily with palatable bait, maintained at a water temperature of 25 ± 0.5 °C, and ensured a dissolved oxygen level of 7.0 ± 0.5 mg·L^−1^ and a pH of 7.5 ± 0.5.

*A. hydrophila* were provided by the Hunan Fisheries Science Research Institute. The acclimatized Chinese soft-shelled turtles were then randomly divided into an experimental group and a control group, with each group containing 300 turtles. Based on preliminary experiments, the median lethal concentration (LC_50_) of *A. hydrophila* for Chinese soft-shelled turtles was determined to be 3.36 × 10^9^ CFU/mL. The bacterial suspension was diluted with PBS buffer to a concentration of 3.7 × 10^8^ CFU/mL, and 100 μL of this suspension was injected intraperitoneally into 300 turtles in the experimental group using a sterile syringe. The control group of 300 Chinese soft-shelled turtles received an equal volume of PBS buffer via intraperitoneal injection.

The health status of the experimental Chinese soft-shelled turtles was continuously monitored for 14 days, with samples collected on the 4th day post-infection. On the 4th day, turtles that exhibited reduced vitality, redness of the ventral plate, and swelling or congestion of the chest and abdomen were classified as the susceptible group (S). Those without apparent infection symptoms were classified as the resistant group (R), while turtles with indeterminate symptoms were excluded. In preliminary observations, mortality among the Chinese soft-shelled turtles peaked on the 4th day post-infection and then gradually declined, suggesting that turtles without significant symptoms after 4 days were resistant to *A. hydrophila* infection. Therefore, sampling was conducted on the 4th day post-infection. Before sampling, we anesthetized the Chinese soft-shelled turtles by placing them in an MS-222 solution (100 μg/L) for 2 min. After anesthesia, the turtles’ bodies were wiped with 75% ethanol, and spleen tissues were rapidly collected from 3 Chinese soft-shelled turtles each from the susceptible (S), resistant (R), and control (C) groups, all under RNase-free conditions. The spleen samples for histological examination were preserved in 4% formaldehyde for paraffin embedding, sectioning, and hematoxylin-eosin (HE) staining, with the preparation method detailed in reference [25]. The remaining samples were placed in labeled cryovials, immediately frozen in liquid nitrogen, and stored at −80 °C for subsequent experiments.

### 2.2. RNA Extraction and Construction of cDNA Libraries

RNA extraction and sequencing of the spleen tissue from the Chinese soft-shelled turtles were conducted by Shanghai OE Biotech Co., Ltd., Shanghai, China. In the transcriptome, “TS-TC” and “TR-TC” denote the differential expression results for the susceptible and resistant groups, respectively. RNA was extracted from the spleen tissues of the susceptible and resistant Chinese soft-shelled turtles using a tissue-specific RNA extraction kit, following the manufacturer’s protocol. The extracted RNA was evaluated for purity, contamination, and degradation using a spectrophotometer. The RNA concentration was determined with a Qubit 2.0 Fluorometer (Thermo Fisher Scientific, Waltham, MA, USA), and the integrity was assessed with an Agilent 4200 instrument (Agilent Technologies, Santa Clara, CA, USA). A total of 1 μg RNA from nine samples was utilized for library construction according to the NEB Ultra Directional RNA Library Prep Kit for Illumina (NEB, Ipswich, MA, USA) guidelines, followed by sequencing on the Illumina NovaSeq 6000 platform (Illumina, Inc. San Diego, CA, USA) with a PE150 strategy. The quality of the clean data was appraised using FastQC software (0.11.9). The transcript sequences were then assembled using Trinity (version v2.4.0, with parameters set to min kmer cov: 7; min glue: 10; and other parameters default). Post-assembly, the transcript sequences were stored in FASTA format.

### 2.3. Protein Extraction and LC-MS/MS Analysis

The extraction and analysis of spleen tissue proteins from Chinese soft-shelled turtles were conducted by Shanghai OE Biotech Co., Ltd., Shanghai, China. In the proteome, PS-PC and PR-PC denote the differential expression results for the susceptible and resistant groups, respectively. Sufficient tissue samples were collected, and the total protein was extracted using ultrasonic lysis. The quality of the protein extraction was assessed, and a portion was used to measure the protein concentration using the BCA (bicinchoninic acid) protein assay method. Additionally, the integrity of the proteins was evaluated using SDS-PAGE. Specific steps were carried out following reference [26]. A separate portion was used for trypsin digestion and labeling; 50 μg of protein from each sample was taken and diluted with lysis buffer to adjust to the same concentration and volume across different groups, and digestion was performed using Trypsin-TPCK, followed by labeling with TMT (tandem mass tags) from Thermo Fisher Scientific (Waltham, MA, USA). Subsequently, equal amounts of each labeled sample were mixed and subjected to reverse-phase chromatographic separation, and the samples were analyzed by LC–MS/MS (Thermo Fisher Scientific, Waltham, MA, USA).

### 2.4. Bioinformatic Analysis

The transcriptome data were aligned using the Bowtie2 software (2.3.3.1) with the parameters set for local-sensitive local alignment. TransDecoder software (5.5.0) was employed to predict the coding sequences and related amino acid sequences of the unigenes. The expression levels of mRNA were normalized to FPKM values, followed by differential analysis using edgeR. In the detection of differentially expressed genes (DEGs), a fold change of 2 or greater and a false discovery rate of 0.001 or less were set as the screening criteria. The *p*-values for significant differences were adjusted using the BH (Benjamini–Hochberg) method. The DEGs were subjected to GO (gene ontology) analysis, encompassing cellular processes, biological components, and molecular functions. KEGG (Kyoto Encyclopedia of Genes and Genomes) enrichment analysis was also performed on the DEGs, with pathways considered enriched if the *p*-value was 0.05 or less.

The proteome data were analyzed using Proteome Discoverer 2.4 software from Thermo Fisher Scientific (Waltham, MA, USA). After obtaining raw data through database searching, credible proteins were selected based on the criteria of a SEQUEST HT score greater than 0 and the presence of at least one unique peptide, excluding any blank values. For the identified proteins, annotation information was extracted from databases like UniProt, KEGG, GO, and KOG/COG to explore their functions. Following the identification of differentially expressed proteins (DEPs), both GO and KEGG enrichment analyses were performed to describe their functional roles. The STRING database was utilized to analyze the DEPs for the species of interest or related species (with a BLAST E-value of 1 × 10^−10^) in order to determine the interaction relationships among the DEPs.

### 2.5. Correlation Analysis of Transcriptome and Proteome

In this study, a transcriptome database was used for protein identification. At the transcript level, the threshold for significant differential expression was set to an absolute fold change (FC) of at least 2 and a false discovery rate (FDR) of no more than 0.001. At the protein level, the threshold for significant differential expression was set to a fold change of at least 1.5 and a *p*-value of no more than 0.05. To explore the correlation between the transcriptome and proteome, the Pearson correlation coefficient was utilized to analyze their relationship. The identified correlational differentially expressed genes and proteins (cor-DEGs-DEPs) were then subjected to KEGG analysis.

### 2.6. Quantitative Real Time PCR (qRT-PCR)

To verify the reliability of the transcriptome data, eight DEGs were selected from each of the TR-TC and TS-TC groups for qRT-PCR analysis. Gene-specific primers were designed using NCBI Primer, and the total RNA was extracted. Subsequently, first-strand cDNA was synthesized and amplified. Detection was performed using the qRT-PCR instrument, qTOWER 2.0 (Analytik Jena AG, Jena, Germany). The fluorescence intensity in each sample’s three biological replicates was measured based on the threshold cycle (Ct) value, and the differences were converted into fold changes using a relative quantification method. The primer sequences used can be found in Supplementary Material Table S1.

## 3. Results

### 3.1. Symptoms in Chinese Soft-Shelled Turtles after A. hydrophila Infection

The Chinese soft-shelled turtles exhibited varying symptoms four days post-infection with *A. hydrophila*. The asymptomatic group exhibited no abnormalities on the body surface, nor were any significant symptoms observed in their internal organs. In contrast, the symptomatic group displayed distinct pathological characteristics, including redness of the plastron (Figure S1A); upon dissection, swelling and redness of the spleen (Figure S1B), as well as congestion of the intestines (Figure S1C) were observed.

### 3.2. Pathological Analysis of the Chinese Soft-Shelled Turtles’ Spleens

**Figure 1:**
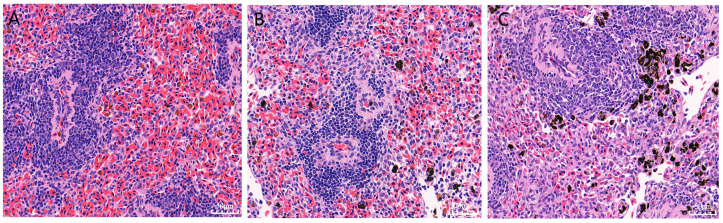
HE-stained sections of spleen tissues from the control, resistant, and susceptible groups of Chinese soft-shelled turtles on the 4th day post-infection with *A. hydrophila*. (**A**) Control group (Group C); (**B**) resistant group (Group R); (**C**) susceptible group (Group S). Note: The scale bar represents 10 μm.

In the spleen tissues of the control and resistant groups of Chinese soft-shelled turtles, the splenic corpuscles and lymphocytes in the diffuse lymphoid tissue were tightly arranged and had abundant capillaries, with the red pulp, white pulp, and marginal zone clearly distinguished (Figure 1A,B). In contrast, the splenic corpuscles in the susceptible group showed a relatively sparse cell arrangement, accompanied by pathological changes, such as increased inflammatory infiltration and hemosiderin deposition in the tissue (Figure 1C).

### 3.3. Transcriptomic Analysis

#### 3.3.1. Illumina Sequencing Results

After constructing and sequencing the libraries derived from the spleen tissue samples of nine Chinese soft-shelled turtles, an average of 100.10 million raw reads were obtained for the control group, 106.32 million for the susceptible group, and 110.81 million for the resistant group. Subsequent filtering ensured that more than 95% of the raw reads in each group met the quality criteria and were thus retained as clean reads. Furthermore, over 78% of the mapped reads in each group achieved the mapping standards. Notably, the Q30 score for all the samples exceeded 95%, demonstrating excellent sequencing quality (Table 1).

#### 3.3.2. Identification of DEGs

**Figure 2:**
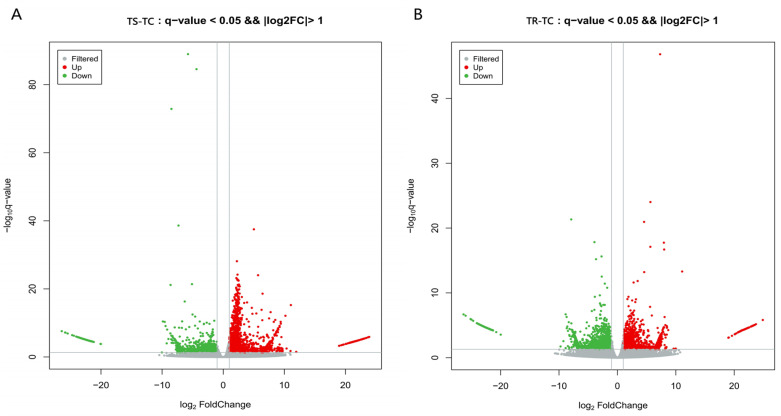
Volcano maps of significant DEGs. (**A**) TS-TC group; (**B**) TR-TC group. Note: Red dots represent upregulated DEGs; green dots represent downregulated DEGs.

A total of 4343 significant DEGs were identified in the TS-TC group, which is notably higher than the 3946 in the TR-TC group. Among them, the number of upregulated genes in the TS-TC group was 2895, exceeding the 1735 in the TR-TC group, and the upregulated genes showed relatively low variability in the expression levels among the samples. Conversely, the TS-TC group had 1448 downregulated genes, which is lower than the 2211 in the TR-TC group, with the downregulated genes in the TR-TC group exhibiting relatively low variability in the expression levels among the samples (Figure 2).

#### 3.3.3. GO and KEGG Enrichment Analyses of DEGs

**Figure 3:**
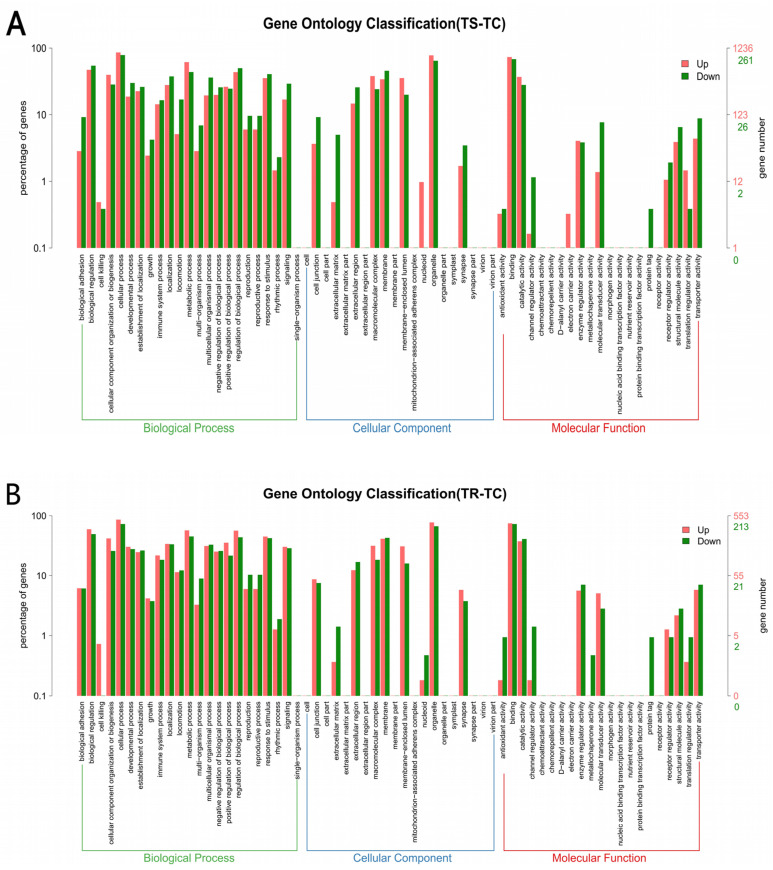
GO classification statistics of DEGs. (**A**) TS-TC group; (**B**) TR-TC group. Note: Red bars represent the enrichment of upregulated DEGs at GO Level II. Green bars represent the enrichment of downregulated DEGs at GO Level II. The x-axis denotes the category names, and the y-axis denotes the number of genes and their corresponding percentage for each category.

The GO functional analysis of DEGs encompasses three primary functional categories at Level I: cellular process, biological component, and molecular function (Figure 3). There are significant differences in the ranking order of the number of DEGs present in various secondary functional categories at Level II between the TS-TC group and the TR-TC group, with the TS-TC group consistently exhibiting a higher number of DEGs compared to the TR-TC group. Specifically, within the biological process category, the TS-TC group showed a higher number of DEGs related to cell division, cell cycle, and mitotic cell cycle. In contrast, the TR-TC group had a higher number of DEGs associated with cell division, specific cell cycle-related signaling pathways (with 39 DEGs), and defense response to virus. In terms of cellular component functions, the TS-TC group had a higher abundance of DEGs related to the nucleolus, extracellular region, and microtubule. Meanwhile, the TR-TC group exhibited a higher number of DEGs linked to the microtubule, midbody, and condensed chromosome kinetochore. For molecular functions, the TS-TC group had a significant number of DEGs involved in microtubule binding, antigen binding, and ATPase activity. On the other hand, the TR-TC group showed a higher prevalence of DEGs related to microtubule binding, ATPase activity, and microtubule motor activity.

**Figure 4:**
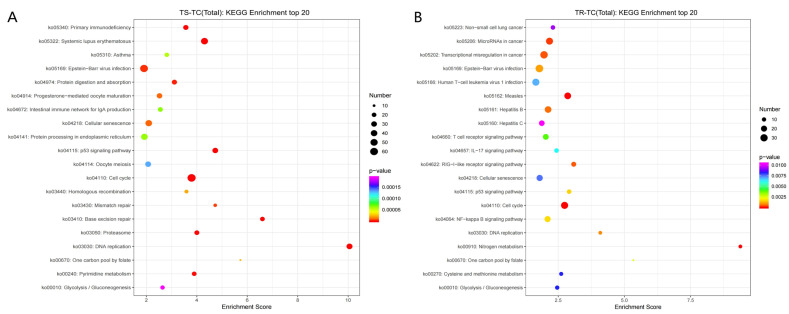
KEGG enrichment analysis. (**A**) TS-TC group; (**B**) TR-TC group. Note: The size of the dots represents the number of DEGs; the color of the dots represents the *p*-value. The x-axis denotes the enrichment score.

The KEGG enrichment analysis of the DEGs revealed the top 20 significantly enriched signaling pathways, as shown in Figure 4. In the TS-TC group, the cell cycle signaling pathway had the highest number of significantly enriched DEGs with 65. According to the enrichment score from high to low, the signaling pathways and their numbers of DEGs are as follows: 33 for DNA replication, 65 for the cell cycle, 46 for systemic lupus erythematosus, and 30 for the p53 signaling pathway. For immune-related pathways, the TS-TC group showed significant enrichment of the DEGs in Fc γ R-mediated phagocytosis and the B cell receptor signaling pathway. In the TR-TC group, Epstein–Barr virus infection had the highest number of enriched DEGs with 33. The other signaling pathways with decreasing enrichment factor included specific cell cycle-related signaling pathways, nitrogen metabolism, and measles. Regarding immune-related pathways, the TR-TC group exhibited significant enrichment of DEGs in the RIG-I-like receptor signaling pathway, chemokine signaling pathway, T cell receptor signaling pathway, and IL-17 signaling pathway.

The summary of DEGs in the TS-TC group can be found in Supplementary Material Table S2. Within the B cell receptor signaling pathway, the DEGs SHIP2, PIK3AP1, CARD11, and IGH are all upregulated (Table S3). The summary of DEGs in the TR-TC group is provided in Supplementary Material Table S4. In the T cell receptor signaling pathway, the DEGs CTLA4, FOS, MAP3K8, CD3E, CD45, NFATC1, VAV, and CARD11 are all upregulated (Table S5); in the RIG-I-like receptor signaling pathway, TRAF3 and USLP2 are significantly upregulated, while IRF7, ISG15, IFIH1, and TRIM25 are downregulated; in the IL-17 signaling pathway, GSK3B and MAPK4/6 are downregulated, whereas TNFAIP3, TRAF3, HSP90A, FOS, and CCL20 are all upregulated; and in the chemokine signaling pathway, FOXO3, ROCK1, PXN, and GSK3B are downregulated, while PRKCB, RAPIA, HRAS, and DOCK2 are all upregulated.

### 3.4. Proteomic Analysis

#### 3.4.1. Identification of DEPs

In the proteome, “PS-PC” and “PR-PC” represent the differential expression results for the susceptible and resistant groups, respectively. Using iTRAQ sequencing technology, a total of 7545 proteins were successfully identified in the Chinese soft-shelled turtles. Subsequently, a further differential expression analysis was conducted on proteins related to *A. hydrophila* infection in the turtles. The results show significant differences in the protein expression post-infection, with 763 proteins in the PS-PC group, of which 576 were upregulated and 187 downregulated; and 568 proteins in the PR-PC group, with 389 upregulated and 179 downregulated.

#### 3.4.2. GO and KEGG Functional Analyses of DEPs

**Figure 5:**
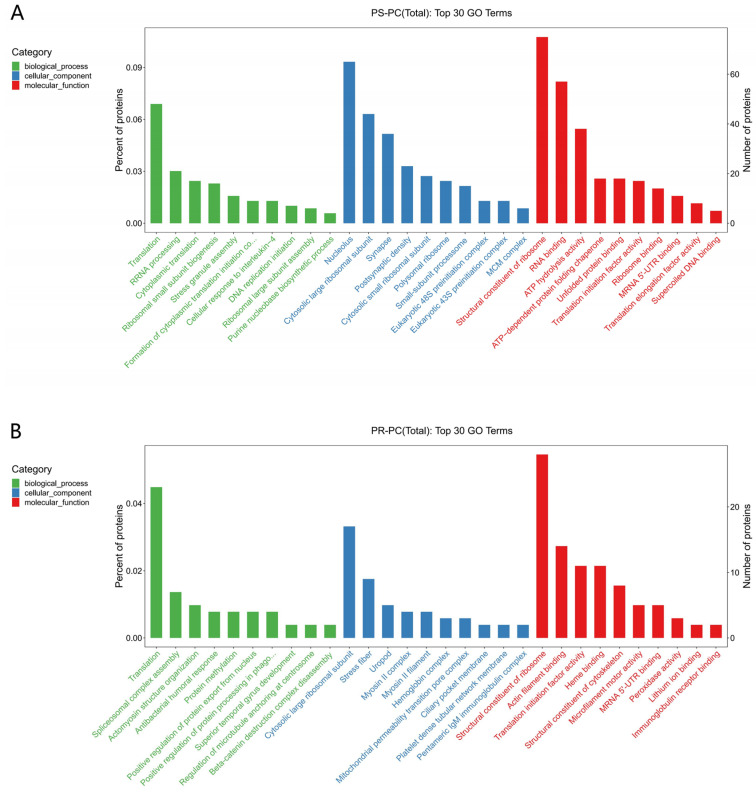
GO function annotation results of DEPs. (**A**) PS-PC group; (**B**) PR-PC group. Note: Green bars represent biological processes; blue bars represent cellular components; red bars represent molecular functions.

The results of the GO functional annotation analysis of DEPs indicate that in the PS-PC group, 697 significantly differential proteins were enriched in 211 biological process terms, 94 cellular component terms, and 106 molecular function terms. In the PR-PC group, 513 differential proteins were enriched in 220 biological process terms, 82 cellular component terms, and 112 molecular function terms. In terms of molecular function enrichment, both the PS-PC and PR-PC groups were most significantly enriched for components of the ribosome’s structure. Regarding biological process enrichment, translation was the most significant term for both groups (Figure 5). However, in terms of cellular component enrichment, there was a difference between the two groups, with the PS-PC group most significantly enriched for the cell nucleus and the PR-PC group for the cytoplasmic large ribosomal subunit.

**Figure 6:**
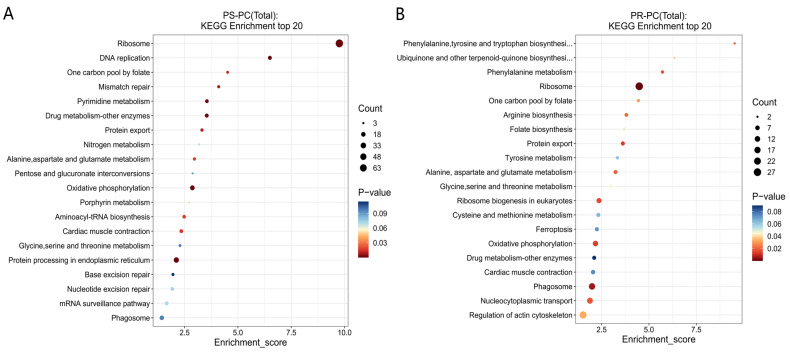
KEGG analysis of significant DEPs. (**A**) PS-PC group; (**B**) PR-PC group. Note: The size of the dots represents the number of DEPs; the color of the dots represents the *p*-value; the x-axis denotes the enrichment score.

The KEGG pathway analysis results indicate that in the PS-PC group, 353 DEPs were significantly enriched in 12 signaling pathways (*p* < 0.05). The primary pathways include the ribosome, DNA replication, oxidative phosphorylation, protein processing in the endoplasmic reticulum, pyrimidine metabolism, mismatch repair, and aminoacyl-tRNA biosynthesis (Figure 6A). In the PR-PC group, 218 DEPs were significantly enriched in 15 signaling pathways (*p* < 0.05). The main pathways include the ribosome, phagosome, oxidative phosphorylation, phenylalanine metabolism, nucleocytoplasmic transport, arginine biosynthesis, and ubiquinone and other terpenoid–quinone biosynthesis (Figure 6B).

In the significantly enriched pathways of the PR-PC group, there is one immune-related pathway, the phagosome, which has enriched 17 proteins, including 11 upregulated and 6 downregulated. No immune-related pathways were found in the PS-PC group (Table S6).

Further analysis of the immune-related pathways indicates that there are some differences in the immune pathways between the PS-PC and PR-PC groups (Tables S7–S9). Both groups are involved in the RIG-I-like receptor signaling pathway, C-type lectin receptor signaling pathway, NOD-like receptor signaling pathway, cytosolic DNA-sensing pathway, and Toll-like receptor signaling pathway.

#### 3.4.3. Differential Protein Interaction Analysis

**Figure 7:**
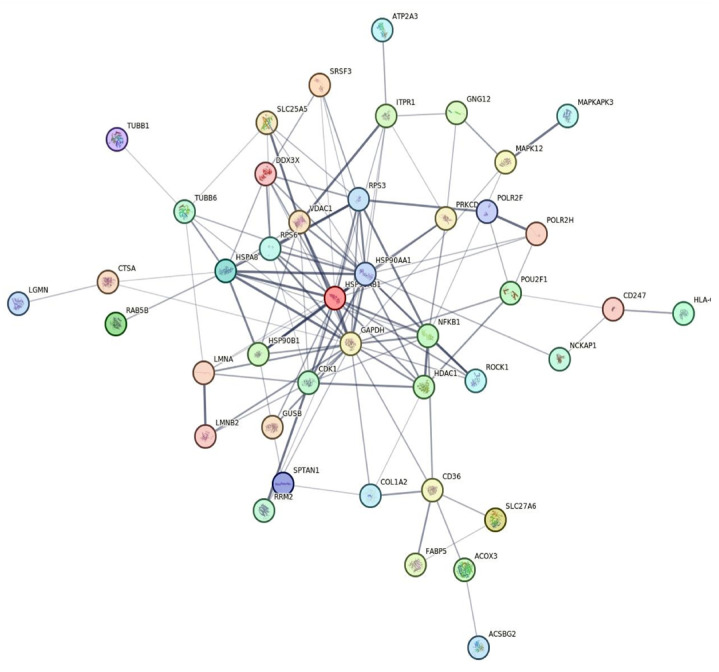
Co-expression network graph of DEPs related to immunity.

A co-expression network of differential immune proteins was established using the STRING database (Figure 7). The coordination among these proteins forms a complex regulatory network. The results show that there are strong interactions between proteins in the spleen of infected Chinese soft-shelled turtles, including GAPDH, AKT1, GSK3B, HSP90AA1, CDK1, HSPA8, HSP90B1, NF-κB1, and HARS. Additionally, proteins such as MAPK12, GNG12, HDAC1, ROCK1, RHOG, RPS3, RPS6, DDX3X, VDAC1, CYBB, NCKAP1, PTGES3, LMNA, and PRCKD are included.

### 3.5. Correlation Analysis of Transcriptome and Proteome

In order to investigate the correlation between the transcriptome and proteome, the Pearson correlation coefficient was used to analyze their relationship, and the results are shown in Figure S2. The overall correlation between the susceptible and resistant groups was found to be relatively poor.

**Figure 8:**
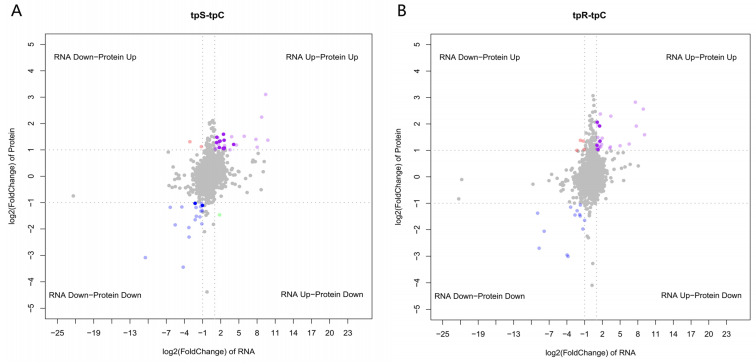
Quadrant plot of cor-DEGs-DEPs. (**A**) tpS-tpC group; (**B**) tpR-tpC group. Note: The gray dots represent no significant differences in gene and protein levels. The purple dots represent upregulation in both the gene and protein. The blue dots represent downregulation in both the gene and protein. The red dots represent downregulation of gene expression with upregulation of protein levels. The green dots represent upregulation of gene expression with downregulation of protein levels.

In the integrated analysis of transcriptomics and proteomics, “tpS-tpC” and “tpR-tpC” represent the differential expression results for the susceptible and resistant groups, respectively. In both the tpS-tpC and tpR-tpC groups, there are 13 and 5 cor-DEGs-DEPs, respectively, that are significantly differentially expressed. In the tpS-tpC group, there are 11 cor-DEGs-DEPs that are significantly upregulated together; 2 cor-DEGs-DEPs that are downregulated together; 1 DEG that is upregulated while the DEP is downregulated; and 2 DEGs that are downregulated while the DEPs are upregulated. In the tpR-tpC group, there are five cor-DEGs-DEPs that are significantly upregulated together and four DEPs that are upregulated while the DEG is downregulated (Figure 8).

**Figure 9:**
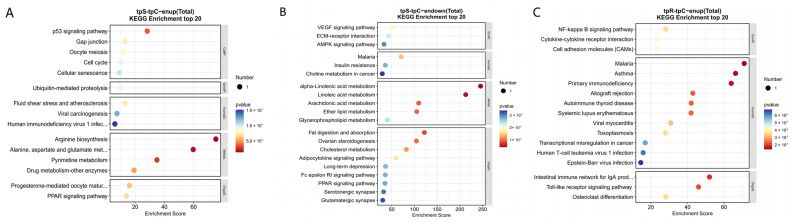
KEGG enrichment analysis of cor-DEGs-DEPs. (**A**) Enriched pathways of cor-DEGs-DEPs upregulated in tpS-tpC group; (**B**) enriched pathways of cor-DEGs-DEPs downregulated in tpS-tpC group; (**C**) Eeriched pathways of cor-DEGs-DEPs upregulated in tpR-tpC group. Note: The size of the dots represents the number of cor-DEGs-DEPs; the color of the dots represents the *p*-value; the x-axis denotes the enrichment score.

The KEGG enrichment analysis indicates that in the tpS-tpC group, 5 cor-DEGs-DEPs are upregulated concurrently across 15 pathways. These genes and proteins are primarily involved in arginine biosynthesis, pyrimidine metabolism, the p53 signaling pathway, and alanine, aspartate, and glutamate metabolism (Figure 9A). Two cor-DEGs-DEPs are downregulated in 32 pathways, with these significant cor-DEGs-DEPs mainly participating in α-linolenic acid metabolism, fat digestion and absorption, and arachidonic acid metabolism (Figure 9B). In the tpR-tpC group, the differentially expressed CD40 gene and protein are upregulated together in the Toll-like receptor signaling pathway, with no common genes and proteins downregulated (Figure 9C).

### 3.6. qRT-PCR Analysis

**Figure 10:**
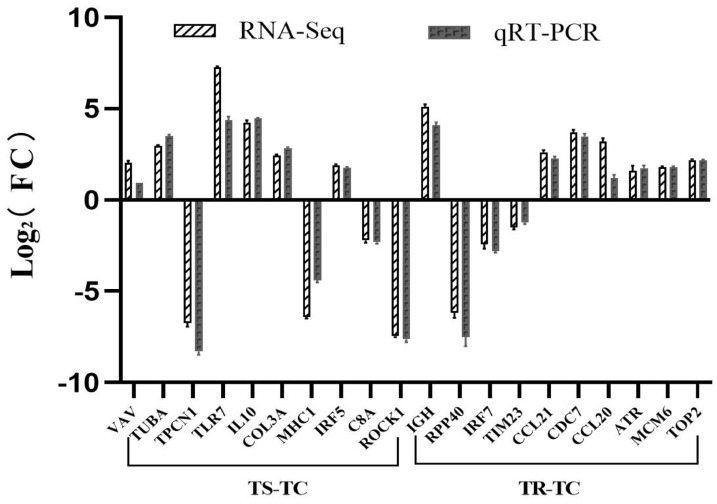
Comparison of relative fold changes between RNA-seq and qRT-PCR results among the TS-TC and TR-TC. Fold changes represented the ratio of gene expression, and β-actin was used as an internal reference. Data are expressed as mean ± SD.

To verify the reliability of the transcriptome data following *A. hydrophila* infection, we randomly selected DEGs from the TS-TC group, including VAV, TUBA, TPCN1, TLR7, IL10, COL3A, MHCI, IRF5, C8A, and ROCK1, and from the TR-TC group, including IGH, RPP40, IRF7, TIM23, CCL21, CDC7, CCL20, ATR, MCM6, and TOP2, for qRT-PCR analysis to assess the consistency of their mRNA expression with the RNA-seq data. These genes are known to be involved in immune response and signal transduction. The validation results showed a high degree of consistency in gene expression trends detected by both RNA-Seq and qRT-PCR, confirming the reliability of the transcriptome data (Figure 10).

## 4. Discussion

Large-scale farming of the Chinese soft-shelled turtle is prone to disease outbreaks, resulting in significant economic losses. Consequently, elucidating the immune defense mechanisms of this species is crucial for rational prevention and control. We conducted sequencing of the transcriptome and proteome of the spleens from Chinese soft-shelled turtles infected with the pathogen *A. hydrophila*. The results reveal that the number of DEGs in the TS-TC group (4667) was significantly higher than that in the TR-TC group (3417). Similarly, the number of differential proteins in the PS-PC group (763) surpassed that of the PR-PC group (568), enabling the identification of numerous immune-related DEGs and proteins in the spleen of the Chinese soft-shelled turtle after infection with *A. hydrophila*.

T cells are crucial immune cells that play an active role in clearing infections from pathogens. When the T cell receptor (TCR) on the surface of T cells binds to the MHC poly-peptide complex (pMHC) carrying antigens, T cells are gradually activated. Following antigen stimulation, the TCR associates with CD4/8, CD3, and CD45. The intracellular domain of CD45 dephosphorylates the inhibitory site of LCK, thereby activating LCK [27]. The CD3 molecule plays an important role in the assembly of the TCR/CD3 complex and the maturation of T cells [28,29], which includes four subunits: γ, δ, ε, and ζ. CD3ε contains an immunoreceptor tyrosine-based activation motif (ITAM motif) [30]. The activated LCK provides a binding site for the tyrosine kinase ZAP70. In this study, CD3ε and CD45 were significantly upregulated in the TR-TC group, promoting the binding of TCR and pMHC, thereby activating the T lymphocytes and signal transduction in the Chinese soft-shelled turtle. The activation of the TCR signaling pathway affects the function of downstream pathways, including the calcium signaling pathway, NF-kB signaling pathway, and MAPK signaling pathway, among others. The calcium signaling pathway activates NFAT, leading to the production of IL-2, which promotes the activation and proliferation of T cells [31]. Ras is essential in the cascade reaction of the MAPK signaling pathway. Once activated, Ras transmits the signal to Raf, MEK1/2, and ERK in the MAPK signaling pathway, initiating the AP-1 transcription factors JUN and FOS to regulate cell growth, survival, proliferation, and differentiation [32,33,34]. In the TR-TC group, Ras and NFAT in the T cell receptor signaling pathway were both significantly upregulated, indicating that both the calcium signaling pathway and the MAPK signaling pathway were activated, effectively promoting the participation of T lymphocytes in the body’s immune response. It can be inferred that the T cell receptor signaling pathway plays an important role in the Chinese soft-shelled turtle’s resistance to *A. hydrophila* infection.

In the PR-PC group, the RIG-I-like receptor signaling pathway, NOD-like receptor signaling pathway, C-type lectin receptor signaling pathway, and Toll-like receptor signaling pathway all activated the NF-κB signaling pathway, leading to the upregulation of NF-κB1 expression. Studies have shown that in the Toll-like receptor signaling pathway, TLR recruits adapter molecules MyD88 and IL-1R-associated kinase, and through signal transduction by TRAF6, TAB2, and TAK1, the NF-κB transcription factor is activated, inducing the production of inflammatory cytokines [35,36,37]. In the NOD-like receptor signaling pathway, TLR19 and TLR22 recognize viral genetic material, initiating the IFN and NF-κB signaling pathways to fend off viral invasion [38,39]. NF-κB, as a family of transcription factors, plays a variety of key roles in regulating immune responses, participating in the modulation of cell activation, cell proliferation, inflammation, and immune responses, among other physiological processes [40]. In this study, the significant upregulation of NF-κB1 protein expression in the PR-PC group suggests that the increased expression of NF-κB1 may enhance the Chinese soft-shelled turtle’s resistance to *A. hydrophila* infection, thereby enabling its survival in challenge experiments.

Phagocytosis is the process by which the host recognizes, engulfs, and destroys invading microorganisms, and it is a fundamental defense mechanism of the innate immune system [41,42]. Phagosomes play an important role in macrophages, participating in tissue reconstruction, clearance of apoptotic cells, and restriction of pathogen spread within cells [43]. The vacuolar membrane proton-translocating ATPase (vATPase) is present in the endomembrane system of all eukaryotic cells, an evolutionarily conserved ATP hydrolysis-driven proton pump responsible for acidifying intracellular organelles [44,45]. In Drosophila, the primary rhodopsin Rh1 is synthesized in the endoplasmic reticulum and transported via the secretory pathway, with the vATPase subunit A playing a key role in the transport of rhodopsin, revealing the conserved function of the vATPase complex in the process of secretory vesicle exocytosis [46]. CTSS is a cysteine protease primarily expressed in antigen-presenting cells, such as macrophages [47], capable of cleaving membrane-bound substrates [48,49,50] and playing an important role in antigen processing and presentation, as well as matrix degradation. MHC class I molecules can recognize and bind to antigenic peptides, and after being transferred outside the cell, they combine with TCR to activate the T cell receptor signaling pathway [51,52]. MHC class I encodes histocompatibility antigens, which are expressed on almost all nucleated cells [53]. Li Wei [54] and others detected MHC class I in the liver, spleen, and kidney of a yellow eel infected with *A. hydrophila*, and its content increased significantly, indicating that MHC is involved in immune regulation. In this study, the proteins vATPase, MHC class I, and CTSS in the phagosome pathway were significantly upregulated, which may be similar to the immune response process in the aforementioned studies, that is, the antigen is first recognized by the receptor MR1 on the surface of phagocytic cells, assisted by vATPases for intracellular transport, and then internalized to form a phagosome. Under the action of the hydrolase CTSS, the protein antigen is degraded into antigenic peptides, combined with MHC class I to form an MHC complex, and then transported outside the cell to combine with TCR, activating T lymphocytes and helping the Chinese soft-shelled turtle to resist bacterial invasion. The significant enrichment of this pathway in the PR-PC group indicates that phagosomes play an important role in the Chinese soft-shelled turtle’s resistance to *A. hydrophila* infection, effectively killing pathogens, triggering inflammatory responses, and activating a series of immune responses. This situation is consistent with the infection of other aquatic animals, where the spleen of black carp (*Mylopharyngodon piceus*) significantly enriched the phagosome pathway 48 h after infection with *A. hydrophila* [55]. In Nile tilapia (*Oreochromis niloticus*), the phagosome pathway was significantly enriched in the spleen and plasma after 15 days of infection with *A. hydrophila* [56].

The quantitative correlation analysis of transcriptomics and proteomics data shows a low correlation coefficient between the two. The KEGG enrichment results indicate that the cor-DEGs-DEPs associated with both transcriptomics and proteomics are involved in the metabolism of plasminogen, glycine, serine, and threonine, as well as the biosynthesis of steroid hormone signaling pathways. This study found that CD40 was significantly upregulated in both transcriptomics and proteomics, revealing that CD40 may play multiple roles in the host immune response [57,58]. CD40 is a type I transmembrane glycoprotein widely expressed on immune cells and tumor cells, closely related to the immune activities of T and B cells [59,60]. CD40L, as the ligand for CD40, produces certain biological functions after binding with CD40 [61,62]. The interaction of CD40–CD40L is an important signal transduction pathway in cellular immunity [57]. In this study, we found that the significant upregulation of IL-10 in the TS-TC group reduced the Chinese soft-shelled turtle’s ability to resist *A. hydrophila*, which may be due to IL-10 inhibiting the activation and maturation of macrophages, thereby suppressing the antigen-specific response of T cells. Studies have shown that the CD40–CD40L co-stimulatory signal can effectively reduce the inhibitory effect of IL-10 on the maturation process of macrophages. The binding of CD40 and CD40L initiates the activation of the NF-κB signaling pathway, leading to a decrease in the expression of IL-10 receptors on the surface of macrophages [63]. There was no significant difference in the expression of IL-10 in the TS-TC group, while CD40 and NF-κB1 were significantly upregulated, suggesting that CD40 may play an important role in inhibiting the expression of IL-10, thereby enhancing the bactericidal immune capacity of the Chinese soft-shelled turtle.

In summary, the DEGs and DEPs were primarily enriched in the TLR, BLR, RIG-I, and phagosome pathways. The synergistic effects of these immune-related pathways may be crucial for enhancing the splenic immune defense of Chinese soft-shelled turtles. Upregulation of immune-related genes, such as CD3ε, CD45, NFAT, and Ras, enhanced T-cell activation and the immune response, which in turn activated the calcineurin pathway and MAPK signaling pathway, improving the immune capability of Chinese soft-shelled turtles. Meanwhile, significant upregulation of CD40 and NF-κB1, potentially by inhibiting IL-10 expression, suggests that CD40 may play a pivotal role in enhancing the antibacterial immune capability of Chinese soft-shelled turtles.

## 5. Conclusions

This study screened for gene and protein expression in the spleen tissues of susceptible and resistant Chinese soft-shelled turtles infected with *A. hydrophila* on the 4th day. The susceptible group exhibited typical pathological changes associated with *A. hydrophila* infection. Significant differences were observed in gene and protein expression and pathway enrichment between the susceptible (S), resistant (R), and control (C) groups. The number of DEGs in the TS-TC group (4667) was significantly higher than that in the TR-TC group (3417), and the number of DEPs in the PS-PC group (763) was significantly higher than that in the PR-PC group (568). DEGs and DEPs were mainly enriched in the TLR, BLR, RIG-I, and phagosome pathways, with the synergistic action of these immune-related pathways potentially crucial for enhancing the spleen’s immune defense in Chinese soft-shelled turtles. The spleen plays an important role in recognizing *A. hydrophila*, activating innate immune responses, and initiating cell activation and inflammatory signal transduction. The results of our study provide new insights into the molecular mechanisms of pathogenesis and resistance in Chinese soft-shelled turtles infected with *A. hydrophila*, which could be utilized to aid in the prevention and treatment of *A. hydrophila* and improve the germplasm breeding of Chinese soft-shelled turtles.

## Figures and Tables

**Table 1:** Sequencing data and quality control results.

Sample	Raw Reads	Clean Reads	Q30 (%)	Mapped Reads (%)
TC1	110.72 M	109.32 M	96.26%	86,248,137 (78.90%)
TC2	86.56 M	85.32 M	95.80%	70,666,788 (82.82%)
TC3	103.01 M	101.71 M	96.25%	85,701,656 (84.26%)
TR1	100.77 M	99.40 M	96.27%	83,549,364 (84.05%)
TR2	116.68 M	115.24 M	96.27%	93,791,300 (81.39%)
TR3	101.50 M	100.30 M	96.31%	82,437,481 (82.19%)
TS1	113.04 M	111.54 M	96.18%	89,351,292 (80.11%)
TS2	112.12 M	110.78 M	96.34%	94,055,975 (84.91%)
TS3	107.27 M	105.89 M	96.22%	86,937,464 (82.10%)

Note: Sample: sample name, TC: transcriptome sample of the control group, TR: transcriptome sample of the resistant group, TS: transcriptome sample of the susceptible group. Raw reads: The total number of sequencing reads obtained directly from the sequencing process, before any data quality control or filtering. Clean reads: The number of sequencing reads that remain after applying data quality control measures, excluding low-quality, contaminated, or otherwise unsuitable reads. Q30 (%): The percentage of bases in the clean reads dataset that have a Phred quality score of at least 30, indicating a base call accuracy of 99.9% or higher. Mapped reads (%): The number and percentage of clean reads that have been successfully aligned to the reference sequences, expressed as a proportion of the total number of clean reads.

## Data Availability

The data presented in the study are deposited in the SequenceRead Archive (https://www.ncbi.nlm.nih.gov/sra), accessed on 11 August 2024, under accession number PRJNA1144239 (http://www.ncbi.nlm.nih.gov/bioproject/1144239), accessed on 11 August 2024. The mass spectrometry proteomics data have been deposited in the ProteomeXchange Consortium (https://proteomecentral.proteomexchange.org) via the iProX partner repository [64,65] with the dataset identifier PXD054656, accessed on 7 August 2024.

## Supplementary Materials

The following supporting information can be downloaded at: https://www.mdpi.com/article/10.3390/genes15101273/s1, Table S1: List of primers used for qPCR. Table S2: DEGs of immune-related pathways in TS-TC group; Table S3: DEGs of B cell receptor signaling pathway; Table S4: DEGs of immune-related pathways in TR-TC group; Table S5: DEGs of T cell receptor signaling pathway; Table S6: DEPs of phagosome signaling pathway; Table S7: Immune-related pathway enrichment; Table S8: DEPs of immune-related pathways in PS-PC group; Table S9: DEPs of immune-related pathways in PR-PC group; Figure S1: Symptoms of Chinese soft-shelled turtles on the 4th day post-infection with *A. hydrophila*. Figure S2: Correlation of associated genes and protein expression changes.
