# Supplementary material for: Transcriptomic and Proteomic Analyses of the Immune Mechanism in Pathogenetic and Resistant Chinese Soft-Shelled Turtle (Pelodiscus sinensis) Infected with Aeromonas hydrophila
Source: Genes (Basel). 2024 Sep 27;15(10):1273. doi: 10.3390/genes15101273 (PMC11508015; doi:10.3390/genes15101273)
Supplement: Supplementary file 1 [file genes-15-01273-s001.zip › genes-3186488-supplementary.pdf]

Supplementary Material

Article title: Transcriptomic and proteomic analyses of the immune mechanism in pathogenetic and resistant Chinese Soft-Shelled Turtle (*Pelodiscus sinensis*) infected with *Aeromonas hydrophila*

Journal name: *Genes*

Author names: Lingrui Ge,Zi'ao Wang,Yazhou Hu,Pei Wang,Qin Qin,Yu Tian, Xiaoqing Wang,Xingxing Wen and Dan Zeng

Table S1 List of Primers Used for qPCR

Primer	Forward primer sequences (5′-3′)	Reverse primer sequences (5′-3′)	Gene ID	Application
VAV	CCAGAAAAAGCCAACGCGAA	TTTAATGGGTCCTGGTCCGC	TRINITY_DN207605_c1_g1_i4_2	qPCR
TUBA	TCCCTTCCTCCGTCCTAGTG	GCTGGCTGGGGAAATAGTGT	TRINITY_DN189635_c1_g3_i2_2	
TPCN1	TTTTGCCAGCAGTGTGATGC	GGCAAGCAGATAAAGGGGGA	TRINITY_DN210720_c0_g2_i1_3	
TLR7	CCACTCTCTGCTGTCGTACC	GAGGTGGAGACCTCCTCAGA	TRINITY_DN196809_c9_g1_i1_1	
IL10	ACGGATTGGCAGCCCAAATA	CACAGCACTGTGGAAAGCAC	TRINITY_DN205506_c8_g1_i3_1	
COL3A	GCCGGGCCTCTCAGAATATC	GGGAGCGATATCCACCACAG	TRINITY_DN211534_c1_g1_i23_1	
MHCI	CCACTCCCTGCGCTATTTCT	GCCCTGTAAGTTCTGGGTCC	TRINITY_DN135398_c5_g1_i3_5	
IRF5	CCAGGACATCTACGCCATCC	TTCTCCTTCGGCTTGTGGTC	TRINITY_DN215802_c1_g1_i4_1	
C8A	GTGAGAAGACCAGACGCCAA	GCACACAAAGTGCCACCATT	TRINITY_DN194051_c0_g1_i13_4	
ROCK1	TGAGGCGCTTGAGTTTCTGT	GCTACACTTACCAAGAGACTGCT	TRINITY_DN144343_c0_g10_i1_5	
IGH	AACACGCGTCATGTTCTTGC	CGGACCCAGTGTAAACCGAA	TRINITY_DN161026_c0_g1_i1_1	
RPP40	TTCCAGCCATTGGGTCACAT	TGTGTCCAGTTCTGGCATCC	TRINITY_DN104765_c0_g1_i1_5	
IRF7	CCTTGAGCAATACGCCATGC	AGTGACCTCGGCAAAGACTG	TRINITY_DN141620_c2_g1_i6_5	
TIM23	CCTGGCGGATAGTGGATGAC	TAGCTGTCAAACTTCCCCGC	TRINITY_DN141529_c9_g1_i1_5	
CCL21	TGAGGGGCACAGCAGTACG	TAGGAGGCGAGCACCTTGAG	TRINITY_DN192630_c0_g1_i5_1	
CDC7	CCTTCTGTCCAACGCTCTGT	CTGGCAGCACCATTGTTCAC	TRINITY_DN192846_c0_g1_i9_3	
CCL20	ACCGGTTTTTCCCACCTGAG	GGTCAGTAAGCACACACCGT	TRINITY_DN166011_c0_g1_i1_2	
ATR	GCAGTGCTAGTCCAGAGGAAT	TGCAGCAATTCGTAGAAGCC	TRINITY_DN214693_c1_g1_i1_1	
MCM6	AACCCGGTCTGTGCCAATAG	ACCAGCCTGAGCTGACTCTA	TRINITY_DN201444_c0_g1_i5_1	
TOP2	TTCAGCCCCACAGTCACATC	CCCTGCTTCCTAGAGGGTCT	TRINITY_DN211406_c0_g1_i12_3	

Table S2 DEGs of immune-related pathways in TS-TC group

KEGG Pathway	Gene Name	NR Annoation	Gene ID	log2FC	*P*-Value
Intestinal immune network for IgA production	TACI	tumor necrosis factor receptor superfamily member 13B	TRINITY_DN107569_c0_g1_i1_4	3.150088112	1.14E-16
	IGH	immunoglobulin heavy chain	TRINITY_DN143395_c0_g1_i4_5	1.478562096	0.000126289
	IGH	immunoglobulin heavy chain	TRINITY_DN161026_c0_g1_i1_1	5.72689823	3.12E-07
	IL10	interleukin 10	TRINITY_DN163787_c0_g1_i1_1	3.808578481	0.000395493
	BCMA	tumor necrosis factor receptor superfamily member 17	TRINITY_DN181595_c0_g1_i2_1	2.600082093	1.34E-05
	ICOS	inducible T-cell co-stimulator	TRINITY_DN189971_c7_g1_i5_2	1.047454094	2.94E-05
	IGH	immunoglobulin heavy chain	TRINITY_DN192723_c0_g1_i1_4	2.16836125	6.43E-11
	IGH	immunoglobulin heavy chain	TRINITY_DN192892_c0_g1_i5_3	2.198002355	4.10E-05
	IGH	immunoglobulin heavy chain	TRINITY_DN193700_c2_g1_i4_2	2.347485596	4.32E-05
	IGH	immunoglobulin heavy chain	TRINITY_DN196388_c1_g1_i4_2	1.609158406	0.000146931
	CD40	tumor necrosis factor receptor superfamily member 5	TRINITY_DN198508_c0_g1_i5_2	1.584503396	5.01E-12
	IGH	immunoglobulin heavy chain	TRINITY_DN200030_c4_g2_i1_4	2.040459771	2.59E-08
	IGH	immunoglobulin heavy chain	TRINITY_DN204885_c0_g2_i1_4	1.271415949	3.17E-06
	IL10	interleukin 10	TRINITY_DN205506_c8_g1_i3_1	3.482787653	1.17E-08
	IL10	interleukin 10	TRINITY_DN205506_c8_g2_i3_1	4.255412757	5.46E-13
	IGH	immunoglobulin heavy chain	TRINITY_DN205542_c4_g1_i16_2	4.041838687	4.66E-05
	TRAV	T cell receptor alpha chain V region	TRINITY_DN206063_c0_g1_i4_2	2.109230125	0.000200315
	IGH	immunoglobulin heavy chain	TRINITY_DN207412_c0_g1_i7_2	1.700563526	2.20E-05
	IGH	immunoglobulin heavy chain	TRINITY_DN208996_c0_g6_i1_4	1.675728252	1.48E-06
	IGH	immunoglobulin heavy chain	TRINITY_DN210703_c0_g1_i3_3	2.338305366	0.000175022
	IGH	immunoglobulin heavy chain	TRINITY_DN213831_c4_g1_i3_3	1.800435096	0.000304511
	IGH	immunoglobulin heavy chain	TRINITY_DN215342_c1_g2_i1_3	2.360095645	9.24E-07
Fc gamma R-mediated phagocytosis	IGH	immunoglobulin heavy chain	TRINITY_DN143395_c0_g1_i4_5	1.478562096	0.000126289
	IGH	immunoglobulin	TRINITY_DN16102	5.72689823	3.12E-07

KEGG Pathway	Gene Name	NR Annoation	Gene ID	log2FC	*P*-Value
		heavy chain	6_c0_g1_i1_1		
	NCF1	neutrophil cytosolic factor 1	TRINITY_DN191642_c3_g1_i2_2	1.03168629	0.000416636
	IGH	TRINITY_DN192892_c0_g1_i5_3	TRINITY_DN192723_c0_g1_i1_4	2.16836125	6.43E-11
	IGH	immunoglobulin heavy chain	TRINITY_DN192892_c0_g1_i5_3	2.198002355	4.10E-05
	IGH	immunoglobulin heavy chain	TRINITY_DN193700_c2_g1_i4_2	2.347485596	4.32E-05
	IGH	immunoglobulin heavy chain	TRINITY_DN196388_c1_g1_i4_2	1.609158406	0.000146931
	CPLA2	cytosolic phospholipase A2 [EC：3.1.1.4]	TRINITY_DN198271_c2_g2_i2_4	-3.327106385	0.000378712
	PTPRC	receptor-type tyrosine-protein phosphatase C [EC：3.1.3.48]	TRINITY_DN199032_c5_g1_i1_2	8.143508307	4.83E-09
	IGH	immunoglobulin heavy chain	TRINITY_DN200030_c4_g2_i1_4	2.040459771	2.59E-08
	PRKCD	novel protein kinase C delta type [EC：2.7.11.13]	TRINITY_DN201685_c1_g1_i3_4	1.461879063	9.42E-11
	PTPRC	receptor-type tyrosine-protein phosphatase C [EC：3.1.3.48]	TRINITY_DN201967_c2_g5_i1_1	9.015981527	4.59E-12
	PTPRC	receptor-type tyrosine-protein phosphatase C [EC：3.1.3.48]	TRINITY_DN201967_c2_g5_i1_1	9.216958039	2.86E-13
	PRKCD	novel protein kinase C delta type [EC：2.7.11.13]	TRINITY_DN201967_c2_g9_i1_1	2.010471039	0.00025632
	IGH	immunoglobulin heavy chain	TRINITY_DN203611_c4_g3_i3_2	1.271415949	3.17E-06
	IGH	immunoglobulin heavy chain	TRINITY_DN204885_c0_g2_i1_4	4.041838687	4.66E-05
	PTPRC	receptor-type tyrosine-protein phosphatase C [EC：3.1.3.48]	TRINITY_DN205542_c4_g1_i16_2	-8.472898759	7.11E-79
	IGH	immunoglobulin heavy chain	TRINITY_DN206608_c0_g1_i1_4	1.700563526	2.20E-05
	VAV	guanine nucleotide exchange factor VAV	TRINITY_DN207412_c0_g1_i7_2	1.014804411	1.24E-05
	VAV	guanine nucleotide exchange factor VAV	TRINITY_DN207605_c1_g1_i4_2	1.949629927	6.69E-05
	ARPC5	actin related	TRINITY_DN20766	1.620741492	2.94E-06

KEGG Pathway	Gene Name	NR Annoation	Gene ID	log2FC	*P*-Value
		protein 2/3 complex， subunit 5	0_c0_g1_i9_1		
	IGH	immunoglobulin heavy chain	TRINITY_DN208203_c0_g1_i2_1	1.675728252	1.48E-06
	IGH	immunoglobulin heavy chain	TRINITY_DN208996_c0_g6_i1_4	2.338305366	0.000175022
	NCF1	neutrophil cytosolic factor 1	TRINITY_DN210703_c0_g1_i3_3	1.018941788	3.75E-06
	PTPRC	receptor-type tyrosine-protein phosphatase C [EC：3.1.3.48]	TRINITY_DN212300_c2_g1_i4_1	3.796343519	1.13E-09
	IGH	immunoglobulin heavy chain	TRINITY_DN213399_c0_g1_i1_3	1.800435096	0.000304511
	SHIP2	phosphatidylinositol-3，4，5-trisphosphate 5-phosphatase 2 [EC：3.1.3.86]	TRINITY_DN213831_c4_g1_i3_3	1.181597468	4.75E-08
	IGH	immunoglobulin heavy chain	TRINITY_DN213907_c2_g1_i12_1	2.360095645	9.24E-07
	IGH	immunoglobulin heavy chain	TRINITY_DN215342_c1_g2_i1_3	1.478562096	0.000126289
	IGH	immunoglobulin heavy chain	TRINITY_DN143395_c0_g1_i4_5	1.478562096	0.000126289
	IGH	immunoglobulin heavy chain	TRINITY_DN192723_c0_g1_i1_4	2.16836125	6.43E-11
	IGH	immunoglobulin heavy chain	TRINITY_DN192892_c0_g1_i5_3	2.198002355	4.10E-05
	IGH	immunoglobulin heavy chain	TRINITY_DN193700_c2_g1_i4_2	2.347485596	4.32E-05
	IGH	immunoglobulin heavy chain	TRINITY_DN196388_c1_g1_i4_2	1.609158406	0.000146931
B cell receptor signaling pathway	DAPP1	dual adapter for phosphotyrosine and 3-phosphotyrosine and 3-phosphoinositide	TRINITY_DN197902_c3_g3_i7_3	1.270830741	3.02E-06
	IGH	immunoglobulin heavy chain	TRINITY_DN200030_c4_g2_i1_4	2.040459771	2.59E-08
	IGH	immunoglobulin heavy chain	TRINITY_DN204885_c0_g2_i1_4	1.271415949	3.17E-06
	IGH	immunoglobulin heavy chain	TRINITY_DN205542_c4_g1_i16_2	4.041838687	4.66E-05
	PIK3AP1	phosphoinositide 3-kinase adapter protein 1	TRINITY_DN207113_c4_g2_i3_2	1.008517046	8.83E-05
	IGH	immunoglobulin heavy chain	TRINITY_DN207412_c0_g1_i7_2	1.700563526	2.20E-05
	VAV	guanine nucleotide exchange factor VAV	TRINITY_DN207605_c1_g1_i4_2	1.014804411	1.24E-05

KEGG Pathway	Gene Name	NR Annoation	Gene ID	log2FC	*P*-Value
	VAV	guanine nucleotide exchange factor VAV	TRINITY_DN207660_c0_g1_i9_1	1.949629927	6.69E-05
	IGH	immunoglobulin heavy chain	TRINITY_DN208996_c0_g6_i1_4	1.675728252	1.48E-06
	NF-κBIE	NF-kappa-B inhibitor epsilon	TRINITY_DN209491_c0_g1_i1_1	1.260458342	9.67E-06
	NF-κBIE	NF-kappa-B inhibitor epsilon	TRINITY_DN209491_c0_g2_i1_1	1.390534189	0.000154403
	IGH	immunoglobulin heavy chain	TRINITY_DN210703_c0_g1_i3_3	2.338305366	0.000175022
	IGH	immunoglobulin heavy chain	TRINITY_DN213831_c4_g1_i3_3	1.800435096	0.000304511
	SHIP2	phosphatidylinositol-3，4，5-trisphosphate 5-phosphatase 2 [EC：3.1.3.86]	TRINITY_DN213907_c2_g1_i12_1	1.181597468	4.75E-08
	CARD11	caspase recruitment domain-containing protein 11	TRINITY_DN214107_c0_g1_i14_1	1.624370128	5.75E-14
	NF-κBIE	NF-kappa-B inhibitor epsilon	TRINITY_DN215668_c2_g2_i1_3	1.148603322	3.89E-06

Table S3 DEGs of B cell receptor signaling pathway

KEGG Pathway	Gene Name	NR Annoation	Gene ID	log2FC	pValue
B cell receptor signaling pathway	IGH	immunoglobulin heavy chain	TRINITY_DN143395_c0_g1_i4_5	1.478562096	0.000126289
	IGH	immunoglobulin heavy chain	TRINITY_DN192723_c0_g1_i1_4	2.16836125	6.43E-11
	IGH	immunoglobulin heavy chain	TRINITY_DN192892_c0_g1_i5_3	2.198002355	4.10E-05
	IGH	immunoglobulin heavy chain	TRINITY_DN193700_c2_g1_i4_2	2.347485596	4.32E-05
	IGH	immunoglobulin heavy chain	TRINITY_DN196388_c1_g1_i4_2	1.609158406	0.000146931
	DAPP1	dual adapter for phosphotyrosine and 3-phosphotyrosine and 3-phosphoinositide	TRINITY_DN197902_c3_g3_i7_3	1.270830741	3.02E-06
	IGH	immunoglobulin heavy chain	TRINITY_DN200030_c4_g2_i1_4	2.040459771	2.59E-08
	IGH	immunoglobulin heavy chain	TRINITY_DN204885_c0_g2_i1_4	1.271415949	3.17E-06
	IGH	immunoglobulin heavy chain	TRINITY_DN205542_c4_g1_i16_2	4.041838687	4.66E-05
	PIK3AP1	phosphoinositide 3-kinase adapter protein 1	TRINITY_DN207113_c4_g2_i3_2	1.008517046	8.83E-05
	IGH	immunoglobulin heavy chain	TRINITY_DN207412_c0_g1_i7_2	1.700563526	2.20E-05
	VAV	guanine nucleotide exchange factor VAV	TRINITY_DN207605_c1_g1_i4_2	1.014804411	1.24E-05
	VAV	guanine nucleotide exchange factor VAV	TRINITY_DN207660_c0_g1_i9_1	1.949629927	6.69E-05
	IGH	immunoglobulin heavy chain	TRINITY_DN208996_c0_g6_i1_4	1.675728252	1.48E-06
	NF-κBIE	NF-kappa-B inhibitor epsilon	TRINITY_DN209491_c0_g1_i1_1	1.260458342	9.67E-06
	NF-κBIE	NF-kappa-B	TRINITY_DN20949	1.39053	0.00015440

KEGG Pathway	Gene Name	NR Annoation	Gene ID	log2FC	pValue
		inhibitor epsilon	1_c0_g2_i1_1	4189	3
	IGH	immunoglobulin heavy chain	TRINITY_DN210703_c0_g1_i3_3	2.338305366	0.000175022
	IGH	immunoglobulin heavy chain	TRINITY_DN213831_c4_g1_i3_3	1.800435096	0.000304511
	SHIP2	phosphatidylinositol-3，4，5-trisphosphate 5-phosphatase 2 [EC：3.1.3.86]	TRINITY_DN213907_c2_g1_i12_1	1.181597468	4.75E-08
	CARD11	caspase recruitment domain-containing protein 11	TRINITY_DN214107_c0_g1_i14_1	1.624370128	5.75E-14

Table S4 DEGs of immune-related pathways in TR-TC group

KEGG Pathway	Gene Name	NR Annoation	Gene ID	log2FC	*P*-Value
RIG-I-like receptor signaling pathway	IRF7	Interferon regulatory factor 3	TRINITY_DN141620_c2_g1_i6_5	-0.131233742	2.51E-07
	IRF7	interferon regulatory factor 7	TRINITY_DN141620_c2_g2_i1_5	-0.136297779	0.013766235
	IRF7	interferon regulatory factor 7	TRINITY_DN141620_c2_g5_i1_5	-0.203162677	0.046353575
	TRAF3	TNF receptor-associated factor 3 isoform X1	TRINITY_DN142404_c0_g3_i4_5	2.271025502	0.016361708
	DHX58	LOW QUALITY PROTEIN：probable ATP-dependent RNA helicase DHX58	TRINITY_DN143998_c1_g4_i2_5	-0.319590285	0.025654734
	CYLD	LOW QUALITY PROTEIN：ubiquitin carboxyl-terminal hydrolase CYLD	TRINITY_DN194661_c0_g1_i9_4	2.201107991	0.00774643
	TRIM25	E3 ubiquitin/ISG15 ligase TRIM25 isoform X1	TRINITY_DN198089_c1_g1_i2_1	-0.361471662	0.020038099
	DDX3X	PREDICTED：ATP-dependent RNA helicase DDX3X-like	TRINITY_DN205126_c2_g4_i1_2	-0.495062763	0.039010243
	ISG15	ubiquitin-like protein ISG15	TRINITY_DN206508_c0_g1_i3_1	-0.130658094	5.94E-05
	ISG15	ubiquitin-like protein ISG15	TRINITY_DN197292_c0_g1_i8_2	-0.160491772	0.003859914
	IFIH1	interferon-induced helicase C domain-containing protein 1	TRINITY_DN213005_c2_g3_i1_1	-0.359137121	0.029604687
	IFIH1	interferon-induced helicase C domain-containing protein 1	TRINITY_DN213005_c2_g5_i1_1	-0.189505571	0.017064796
	IFIH1	interferon-induced helicase C domain-containing protein 1	TRINITY_DN143298_c3_g5_i1_5	-0.342396255	0.003221548
T cell receptor signaling pathway	CTLA4	cytotoxic T-lymphocyte protein 4	TRINITY_DN171844_c0_g1_i7_2	8.838423826	3.28E-05
	FOS	proto-oncogene c-Fos	TRINITY_DN189433_c0_g1_i2_1	2.924477248	0.003442506
	MAP3K8	Mitogen-activated protein kinase kinase kinase 8	TRINITY_DN199535_c2_g4_i1_2	2.55229648	0.039015228
	HRAS	GTPase HRas isoform X1	TRINITY_DN199560_c0_g1_i3_3	3.185901417	0.040404034
	CD3E	T-cell surface glycoprotein CD3 epsilon chain precursor	TRINITY_DN200275_c2_g1_i7_2	2.360063621	0.014179893
	PTPRC	receptor-type tyrosine-protein phosphatase C isoform X4	TRINITY_DN201967_c2_g5_i1_1	1665493.395	0.000120763

KEGG Pathway	Gene Name	NR Annoation	Gene ID	log2FC	*P*-Value
IL-17 signaling pathway	PTPRC	receptor-type tyrosine-protein phosphatase C isoform X3	TRINITY_DN201967_c2_g9_i1_1	2433046.892	6.79E-05
	PTPRC		TRINITY_DN210397_c7_g2_i1_3	2.917150478	0.009794549
	PTPRC	receptor-type tyrosine-protein phosphatase C isoform X3	TRINITY_DN213399_c0_g1_i1_3	11.21211144	0.008796629
	CARD11	caspase recruitment domain-containing protein 11 isoform X3	TRINITY_DN205411_c4_g1_i8_3	2.79762803	0.025094063
	GSK3B	hypothetical protein DNTS_028787	TRINITY_DN206503_c2_g1_i11_1	-0.390522167	0.000823678
	NFATC1	WD repeat-containing protein 75	TRINITY_DN210842_c0_g1_i9_3	2.801971001	0.002065594
	VAV	proto-oncogene vav	TRINITY_DN211935_c0_g2_i1_3	3.405869255	0.011274566
	VAV	guanine nucleotide exchange factor VAV2 isoform X1	TRINITY_DN212873_c3_g2_i3_3	2.103292754	0.017154973
	VAV	proto-oncogene vav	TRINITY_DN207660_c0_g1_i9_1	4.946070981	2.98E-05
	MAP3K14	mitogen-activated protein kinase kinase kinase 14 isoform X1	TRINITY_DN213468_c1_g1_i5_3	2.329503125	0.030569371
	CARD11	caspase recruitment domain-containing protein 11 isoform X3	TRINITY_DN214107_c0_g1_i14_1	2.312677395	0.002862726
	CARD11	caspase recruitment domain-containing protein 11 isoform X3	TRINITY_DN212130_c1_g1_i7_3	3.008145235	9.26E-06
	TNFAIP3,	tumor necrosis factor alpha-induced protein 3 isoform X1	TRINITY_DN140558_c0_g1_i5_5	2.327141813	0.000207863
	TRAF3	TNF receptor-associated factor 3 isoform X1	TRINITY_DN142404_c0_g3_i4_5	2.271025502	0.016361708
	CCL20	C-C motif chemokine 20-like	TRINITY_DN166011_c0_g1_i1_2	48.27997875	1.43E-08
	CCL20	C-C motif chemokine 20-like	TRINITY_DN166011_c0_g2_i1_2	8.34780154	0.007112355
	CCL20	C-C motif chemokine 20-like	TRINITY_DN255005_c0_g1_i1_4	6.659667007	0.000527332
	HSP90A	heat shock protein HSP 90-alpha	TRINITY_DN182679_c0_g1_i3_1	4.300854384	0.005292056
	FOS	proto-oncogene c-Fos	TRINITY_DN189433_c0_g1_i2_1	2.924477248	0.003442506
	GSK3B	hypothetical protein DNTS_028787	TRINITY_DN206503_c2_g1_i11_1	0.390522167	0.000823678
	TNFAIP3	tumor necrosis factor alpha-induced protein 3 isoform X1	TRINITY_DN208810_c2_g1_i9_1	2.902876928	7.84E-06
	HSP90A	Putative heat shock protein HSP 90-beta-3	TRINITY_DN210625_c8_g1_i3_3	5.824490002	3.16E-06
	MAPK4_6	mitogen-activated protein kinase 4 isoform X1	TRINITY_DN36354_c0_g2_i1_4	-3.46E-07	5.35E-05

KEGG Pathway	Gene Name	NR Annoation	Gene ID	log2FC	*P*-Value
	FOXO3	forkhead box protein O3	TRINITY_DN140273_c0_g3_i4_5	0.409197616	0.0007159
	ROCK1	Rho-associated protein kinase 1， partial	TRINITY_DN144343_c0_g10_i1_5	0.005963555	0.000318495
	ROCK1	rho-associated protein kinase 1 isoform X3	TRINITY_DN144343_c0_g2_i17_5	0.426577247	0.012101751
	CCL20	C-C motif chemokine 20-like	TRINITY_DN166011_c0_g1_i1_2	48.27997875	1.43E-08
	CCL20	C-C motif chemokine 20-like	TRINITY_DN166011_c0_g2_i1_2	8.34780154	0.007112355
	CCL20	C-C motif chemokine 20-like	TRINITY_DN255005_c0_g1_i1_4	6.659667007	0.000527332
	CCL21	S-adenosyl-L-homocysteine hydrolase	TRINITY_DN192630_c0_g1_i5_1	5.628178802	3.11E-05
	RAP1A	hypothetical protein CIB84_015959	TRINITY_DN199161_c0_g1_i3_3	3.156514154	0.000868503
	HRAS	GTPase HRas isoform X1	TRINITY_DN199560_c0_g1_i3_3	3.185901417	0.040404034
	PXN	LOW QUALITY PROTEIN： leupaxin	TRINITY_DN204708_c1_g1_i1_1	0.155988601	3.06E-13
	GSK3B	hypothetical protein DNTS_028787	TRINITY_DN206503_c2_g1_i11_1	0.390522167	0.000823678
	JAK3	tyrosine-protein kinase JAK3	TRINITY_DN207255_c0_g1_i10_2	3.803156298	0.01496014
	VAV	proto-oncogene vav	TRINITY_DN207660_c0_g1_i9_1	4.946070981	2.98E-05
	GNAI	guanine nucleotide-binding protein G（i） subunit alpha-2	TRINITY_DN208657_c0_g1_i25_1	8.344590966	2.62E-09
	CCR6	C-C chemokine receptor type 6	TRINITY_DN208666_c2_g1_i2_3	2.916549455	0.008102786
Chemokine signaling pathway	GRK4_5_6	G protein-coupled receptor kinase 5-like isoform X1	TRINITY_DN209741_c1_g1_i18_4	0.22650535	0.000420615
	DOCK2	dedicator of cytokinesis protein 2	TRINITY_DN211016_c1_g9_i1_4	3.277204347	0.003168418
	DOCK2	dedicator of cytokinesis protein 2	TRINITY_DN215539_c3_g4_i1_1	3.326183435	0.006275319
	VAV	proto-oncogene vav	TRINITY_DN211935_c0_g2_i1_3	3.405869255	0.011274566
	VAV	guanine nucleotide exchange factor VAV2 isoform X1	TRINITY_DN212873_c3_g2_i3_3	2.103292754	0.017154973
	PRKCB	protein kinase C beta type	TRINITY_DN215928_c6_g2_i4_3	3.360050542	0.041021623
	PTR2B	protein-tyrosine kinase 2-beta isoform X2	TRINITY_DN215971_c2_g1_i16_3	2.407497253	0.029684381

Table S5 DEGs of T cell receptor signaling pathway

KEGG Pathway	Gene Name	NR Annoation	Gene ID	log2FC	pValue
T cell receptor signaling pathway	CTLA4	cytotoxic T-lymphocyte protein 4	TRINITY_DN171844_c0_g1_i7_2	8.838423826	3.28E-05
	FOS	proto-oncogene c-Fos	TRINITY_DN189433_c0_g1_i2_1	2.924477248	0.003442506
	MAP3K8	Mitogen-activated protein kinase kinase kinase 8	TRINITY_DN199535_c2_g4_i1_2	2.55229648	0.039015228
	HRAS	GTPase HRas isoform X1	TRINITY_DN199560_c0_g1_i3_3	3.185901417	0.040404034
	CD3E	T-cell surface glycoprotein CD3 epsilon chain precursor	TRINITY_DN200275_c2_g1_i7_2	2.360063621	0.014179893
	PTPRC	receptor-type tyrosine-protein phosphatase C isoform X4	TRINITY_DN201967_c2_g5_i1_1	1665493.395	0.000120763
	PTPRC	receptor-type tyrosine-protein phosphatase C isoform X3	TRINITY_DN201967_c2_g9_i1_1	2433046.892	6.79E-05
	PTPRC	receptor-type tyrosine-protein phosphatase C isoform X3	TRINITY_DN213399_c0_g1_i1_3	11.21211144	0.008796629
	CARD11	caspase recruitment domain-containing protein 11 isoform X3	TRINITY_DN205411_c4_g1_i8_3	2.79762803	0.025094063
	GSK3B	hypothetical protein DNTS_028787	TRINITY_DN206503_c2_g1_i11_1	-0.390522167	0.000823678
	NFATC1	WD repeat-containing protein 75	TRINITY_DN210842_c0_g1_i9_3	2.801971001	0.002065594
	VAV	proto-oncogene vav	TRINITY_DN211935_c0_g2_i1_3	3.405869255	0.011274566
	VAV	guanine nucleotide exchange factor VAV2 isoform X1	TRINITY_DN212873_c3_g2_i3_3	2.103292754	0.017154973

KEGG Pathway	Gene Name	NR Annoation	Gene ID	log2FC	pValue
	VAV	proto-oncogene vav	TRINITY_DN207660_c0_g1_i9_1	4.946070981	2.98E-05
	MAP3K14	mitogen-activated protein kinase kinase kinase 14 isoform X1	TRINITY_DN213468_c1_g1_i5_3	2.329503125	0.030569371
	CARD11	caspase recruitment domain-containing protein 11 isoform X3	TRINITY_DN214107_c0_g1_i14_1	2.312677395	0.002862726
	CARD11	caspase recruitment domain-containing protein 11 isoform X3	TRINITY_DN212130_c1_g1_i7_3	3.008145235	9.26E-06

Table S6 DEPs of Phagosome signaling pathway

KEGG Pathway	Proteins Name	NR Annoation	proteins ID	log2FC	*p*-Value
Phagosome	EPX	Eosinophil peroxidase	TRINITY_DN183620_c0_g1_i1_2	-0.313260694	0.010634997
	TUBB1	Tubulin beta chain	TRINITY_DN183564_c0_g1_i1_4	-0.445197585	0.01697708
	MRC2	Mannose receptor C type 2	TRINITY_DN215097_c0_g1_i1_3	-0.335883033	0.032303317
	RAB5B	RAB5B，member RAS oncogene family	TRINITY_DN202046_c1_g1_i3_4	-0.291519365	0.028858024
	EPX	Eosinophil peroxidase	TRINITY_DN197681_c0_g1_i1_1	-0.380636046	0.003841604
	TUBA	Tubulin alpha chain	TRINITY_DN218284_c0_g1_i1_3	-0.309472462	0.018241908
	ITGA5	Integrin subunit alpha 5	TRINITY_DN215903_c0_g1_i13_3	-0.387722935	0.045935808
	M6PR	Mannose-6-phosphate receptor，cation dependent	TRINITY_DN205336_c6_g2_i7_1	0.61718233	0.001079826
	ATP6V1H	V-type proton ATPase subunit H	TRINITY_DN135573_c0_g1_i5_5	0.381976848	0.031985181
	ATP6V0D1	ATPase H+ transporting V0 subunit d1	TRINITY_DN211957_c0_g1_i4_3	0.295006796	0.010837537
	IG	Ig-like domain-containing protein	TRINITY_DN142466_c0_g1_i1_5	0.511757372	0.00637834
	ATP6V1C1	V-type proton ATPase subunit C	TRINITY_DN140195_c2_g1_i5_5	0.466392832	0.026421505
	MRC1	Mannose receptor C-type 1	TRINITY_DN196371_c1_g4_i1_2	0.409358408	0.035503967
	MRC1	Mannose receptor C-type 1	TRINITY_DN206228_c4_g1_i5_2	0.849802815	0.046695663
	MHCI	Ig-like domain-containing protein	TRINITY_DN206517_c3_g1_i18_2	1.001266448	0.01767821
	CTSS	Cathepsin S	TRINITY_DN215444_c4_g2_i1_1	0.904621952	0.026555383
	CYBB	Cytochrome b-245 heavy chain	TRINITY_DN210327_c2_g5_i1_4	0.532577921	0.042317503

Table S7 Immune-related pathway enrichment

Pathway	Map ID	PROTEIN COUNTS			
		PS-PC	*p*-Value	PR-PC	*p*-Value
C-type lectin receptor signaling pathway	pss04625	5	0.98	8	0.58
NOD-like receptor signaling pathway	pss04621	8	0.93	9	0.61
Toll-like receptor signaling pathway	pss04620	1	0.99	4	0.69
RIG-I-like receptor signaling pathway	pss04622	4	0.64	2	0.85
Cytosolic DNA-sensing pathway	pss04623	2	0.89	1	0.94

Table S8 DEPs of immune-related pathways in PS-PC group

KEGG Pathway	Proteins Name	NR Annoation	proteins ID	log2FC	*p*-Value
RIG-I-like receptor signaling pathway	SPTAN1	Spectrin alpha， non-erythrocytic 1	TRINITY_DN141847_c3_g1_i12_5	-0.360187716	0.002410321
	LMNA	Lamin A/C	TRINITY_DN200137_c0_g1_i4_4	-0.393305196	0.013518601
	ACTA1	Actin alpha 1， skeletal muscle	TRINITY_DN210535_c2_g4_i1_3	-0.541630462	0.03119083
Cytosolic DNA-sensing pathway	POLR2H	RNA polymerase II subunit H	TRINITY_DN196067_c0_g2_i3_4	0.288744169	0.016186367
	POLR2F	DNA-directed RNA polymerases I， II， and III subunit RPABC2	TRINITY_DN189638_c0_g1_i2_3	0.407458596	0.013340021
NOD-like receptor signaling pathway	VDAC1	Voltage dependent anion channel 1	TRINITY_DN52404_c0_g1_i1_4	-0.39460478	0.00045305
	MAPK12	mitogen-activated protein kinase	TRINITY_DN209068_c6_g1_i4_3	-0.327271222	0.048358181
	ITPR1	Inositol 1， 4， 5-trisphosphate receptor	TRINITY_DN204290_c2_g1_i7_1	-0.357635188	0.003074988
	HSP90AB1	Heat shock protein 90 alpha family class B member 1	TRINITY_DN139998_c1_g1_i5_5	0.582409579	0.000686924
	HSP90AA1	Heat shock protein 90 alpha family class A member 1	TRINITY_DN182679_c0_g1_i3_1	0.316903181	0.000594092
	PRKCD	Protein kinase C delta type	TRINITY_DN201685_c1_g1_i3_4	0.330819926	0.007610228
	VDAC1	Voltage dependent anion channel 1	TRINITY_DN200123_c0_g1_i8_1	0.364411426	0.005159068
	NLRX1	NLR family member X1	TRINITY_DN206749_c0_g1_i4_4	0.293687912	0.010710378
C-type lectin receptor signaling pathway	CD247	T-cell surface glycoprotein CD3 zeta chain	TRINITY_DN203418_c0_g1_i1_4	-0.473776467	0.028183896
	MAPK12	mitogen-activated protein kinase	TRINITY_DN209068_c6_g1_i4_3	-0.327271222	0.048358181
	ITPR1	Inositol 1， 4， 5-trisphosphate receptor	TRINITY_DN204290_c2_g1_i7_1	-0.357635188	0.003074988

KEGG Pathway	Proteins Name	NR Annoation	proteins ID	log2FC	*p*-Value
Toll-like receptor signaling pathway	PRKCD	Protein kinase C delta type	TRINITY_DN201685_c1_g1_i3_4	0.330819926	0.007610228
	NF-κB1	Nuclear factor NF-kappa-B p105 subunit	TRINITY_DN143087_c0_g1_i1_5	0.445677474	0.00215889
	MAPK12	mitogen-activated protein kinase	TRINITY_DN209068_c6_g1_i4_3	-0.327271222	0.048358181

Table S9 DEPs of immune-related pathways in PR-PC group

KEGG Pathway	Proteins Name	NR Annoation	proteins ID	log2FC	*p*-Value
RIG-I-like receptor signaling pathway	MAPK12	mitogen-activated protein kinase	TRINITY_DN209068_c6_g1_i4_3	-0.299721173	0.025809802
	NF-κB1	Nuclear factor NF-kappa-B p105 subunit	TRINITY_DN202971_c1_g1_i7_2	0.389884759	0.011061427
Cytosolic DNA-sensing pathway	NF-κB1	Nuclear factor NF-kappa-B p105 subunit	TRINITY_DN202971_c1_g1_i7_2	0.389884759	0.011061427
NOD-like receptor signaling pathway	VDAC1	Voltage dependent anion channel 1	TRINITY_DN52404_c0_g1_i1_4	-0.529990529	0.00253173
	ANTXR2	Anthrax toxin receptor	TRINITY_DN141706_c0_g3_i4_5	-0.276559906	0.018822711
	MAPK12	mitogen-activated protein kinase	TRINITY_DN209068_c6_g1_i4_3	-0.299721173	0.025809802
	YWHAE	Tyrosine 3-monooxygenase/tryptophan 5-monooxygenase activation protein epsilon	TRINITY_DN209100_c1_g2_i7_4	0.397944409	0.008964831
	RHOA	Ras homolog family member A	TRINITY_DN193838_c2_g3_i2_4	0.451504144	0.032149991
	PRKCD	Protein kinase C delta type	TRINITY_DN201685_c1_g1_i3_4	0.287173554	0.034227315
	NF-κB1	Nuclear factor NF-kappa-B p105 subunit	TRINITY_DN202971_c1_g1_i7_2	0.389884759	0.011061427
	BRCC3	BRCA1/BRCA2-containing complex subunit 3	TRINITY_DN204891_c2_g1_i2_1	0.728203335	0.041564169
	CYBB	Cytochrome b-245 heavy chain	TRINITY_DN210327_c2_g5_i1_4	0.532577921	0.042317503
C-type lectin receptor signaling pathway	PTPN5	Tyrosine-protein phosphatase non-receptor type	TRINITY_DN141406_c0_g1_i8_5	-0.305898583	0.034564091
	HRAS	HRas proto-oncogene，GTPase	TRINITY_DN200623_c7_g1_i8_4	-0.272543232	0.00967408
	MAPK12	mitogen-activated protein kinase	TRINITY_DN209068_c6_g1_i4_3	-0.299721173	0.025809802
	RHOA	Ras homolog family member A	TRINITY_DN193838_c2_g3_i2_4	0.451504144	0.032149991

KEGG Pathway	Proteins Name	NR Annoation	proteins ID	log2FC	*p*-Value
	PRKCD	Protein kinase C delta type	TRINITY_DN201685_c1_g1_i3_4	0.287173554	0.034227315
	AKT1	RAC-alpha serine/threonine-protein kinase	TRINITY_DN200303_c0_g1_i9_2	0.358720126	0.035994626
	NF-κB1	Nuclear factor NF-kappa-B p105 subunit	TRINITY_DN202971_c1_g1_i7_2	0.389884759	0.011061427
	ARHGEF12	Rho guanine nucleotide exchange factor 12	TRINITY_DN215448_c0_g1_i28_1	0.404011882	0.003726686
Toll-like receptor signaling pathway	MAPK12	mitogen-activated protein kinase	TRINITY_DN209068_c6_g1_i4_3	-0.299721173	0.025809802
	AKT1	RAC-alpha serine/threonine-protein kinase	TRINITY_DN200303_c0_g1_i9_2	0.358720126	0.035994626
	NF-κB1	Nuclear factor NF-kappa-B p105 subunit	TRINITY_DN202971_c1_g1_i7_2	0.389884759	0.011061427
	MAP2K4	Mitogen-activated protein kinase kinase 4	TRINITY_DN198204_c2_g1_i9_2	0.538880998	0.001947334

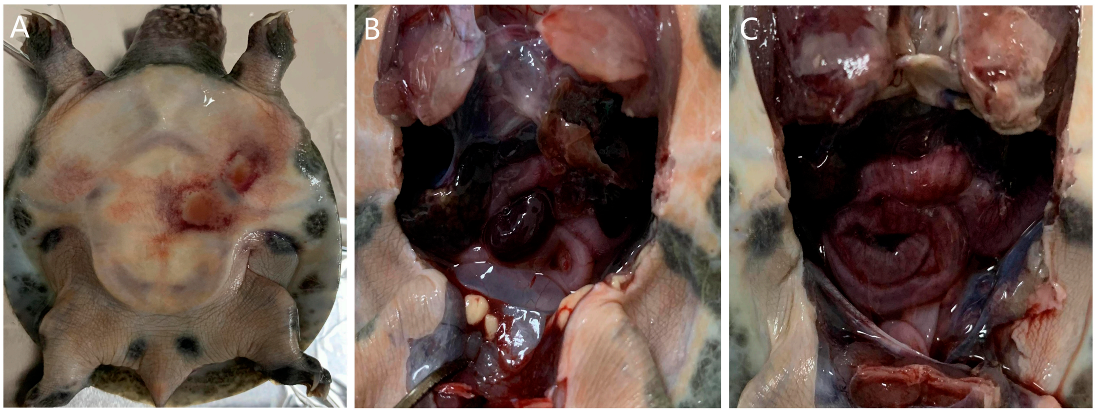

Figure S1. Symptoms of Chinese soft-shelled turtles on the 4th day post-infection with *A. hydrophila*. (A) Reddish underbelly; (B) Swollen and red spleen; (C) Intestinal congestion.

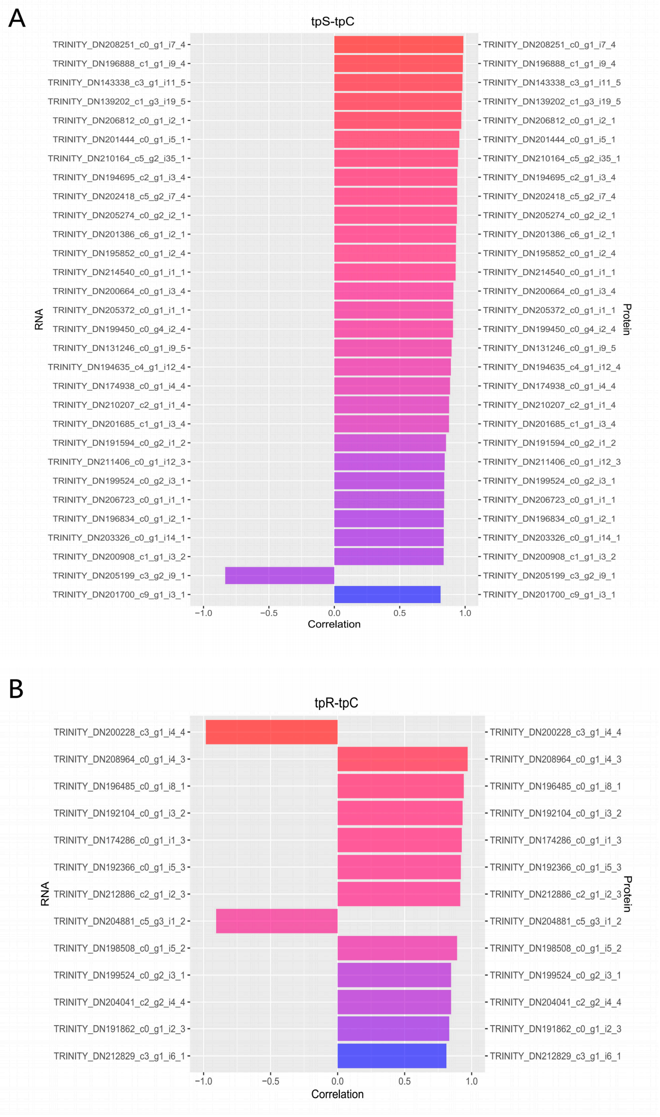

Figure S2. Correlation of associated genes and protein expression changes. (A) tpS-tpC group; (B) tpR-tpC group.
